# The impact of progressive chronic kidney disease on hepatic drug metabolism

**DOI:** 10.1016/j.dmd.2025.100085

**Published:** 2025-04-28

**Authors:** Emily D. Hartjes, Yong Jin Lim, Thomas J. Velenosi, Kait F. Al, Jean M. Macklaim, Andrew S. Kucey, Gregor Reid, Jeremy P. Burton, Gregory B. Gloor, Bradley L. Urquhart

**Affiliations:** 1 Department of Physiology and Pharmacology, Schulich School of Medicine and Dentistry, Western University, London, Ontario, Canada; 2 Faculty of Pharmaceutical Sciences, University of British Columbia, Vancouver, British Columbia, Canada; 3 Department of Microbiology & Immunology, Western University, London, Ontario, Canada; 4 Canadian Centre for Human Microbiome and Probiotics, London, Ontario, Canada; 5 Department of Biochemistry, University of Western Ontario, London, Ontario, Canada; 6 Division of Nephrology, Department of Medicine, Schulich School of Medicine and Dentistry, Western University, London, Ontario, Canada

**Keywords:** Chronic kidney disease, Drug metabolism, Metabolomics, Gut microbiome

## Abstract

Nonrenal clearance pathways such as drug metabolism are decreased in severe chronic kidney disease (CKD). The impact of progression of CKD on hepatic drug metabolism is unknown. We characterized the effect of progressive CKD on hepatic cytochrome P450 expression and evaluated dysbiosis and uremia as kidney function declined. Rats fed control or CKD-inducing adenine diet were studied at 5 time points over 42 days. Cytochrome P450 expression and activity were compared with alterations in the (1) plasma and liver metabolome and (2) gut bacterial microbiota. CYP3A2 and CYP2C11 were downregulated in CKD by ≥76% (*P* < .001) concurrently with or slightly prior to CKD onset as defined by serum creatinine. Metabolite profiles were altered prior to changes in the gut microbiota, and gut-derived uremic toxins including indoxyl sulfate, phenyl sulfate, and 4-ethylphenyl sulfate correlated with CYP3A2 or CYP2C11 expression. Bacterial genera *Turicibacter* and *Parabacteroides* were identified as being characteristic of CKD. In conclusion, CYP3A2 and CYP2C11 are downregulated before dysbiosis and uremia.

**Significance Statement:**

This study describes the effect of progressive kidney disease on hepatic CYP2C11 and CYP3A2 enzyme expression and activity in a rat model of CKD. Expression and activity of drug metabolizing enzymes occurs prior to uremia or dysbiosis.

## 1 Introduction

Chronic kidney disease (CKD) is a progressive and irreversible loss of kidney function over time affecting approximately 10% of the global population (Jadoul et al, 2024). Progressive loss of kidney function in CKD leads to accumulation of waste products in plasma, resulting in uremia (Meyer and Hostetter, 2007). Uremia contributes to the progression of CKD into end-stage renal disease (Meyer and Hostetter, 2007) and has also been associated with the activation of the immune response, gut microbial alterations (Vitetta and Gobe, 2013), and cardiovascular events (Ito and Yoshida, 2014).

The impairment of renal drug clearance in CKD is well established. However, nonrenal, hepatic drug clearance is also significantly decreased in CKD. Nonrenal drug clearance was identified by Kidney Disease: Improving Global Outcomes as an important consideration for dose recommendations (Matzke et al, 2011). Patients with CKD experience altered pharmacokinetics, partially mediated by altered activity of cytochrome P450 drug metabolizing enzymes (Nolin et al, 2008; Ladda and Goralski, 2016). CYP3A4 and CYP2C9 metabolize approximately 43% of all clinically relevant drugs (Zanger and Schwab, 2013). There is evidence that drugs such as warfarin (CYP2C9 substrate) and midazolam (CYP3A4 substrate) have altered pharmacokinetics in CKD, although the mechanism for these observations is controversial (Dreisbach et al, 2003; Nolin et al, 2009; Thomson et al, 2015; Tuey et al, 2022). Previous studies have demonstrated the rat orthologs of CYP3A4 (Cyp3a2) and CYP2C9 (Cyp2c11) have decreased expression and activity in models of CKD (Guévin et al, 2002; Velenosi et al, 2012). Although several studies suggest hepatic drug metabolism is decreased in CKD (Nolin et al, 2008; Ladda and Goralski, 2016), the exact mechanisms underlying cytochrome P450 downregulation are poorly understood. Uremia, hormones, gut bacterial alterations, and associated inflammation are all factors proposed to affect drug metabolizing enzymes in CKD. Despite CKD being a progressive disease, many studies focus exclusively on severe CKD, whereas the majority of patients suffer from earlier stages of the disease. This leaves a gap in our understanding of the temporal relationship between CKD progression and changes in cytochrome P450 expression. Uremia may influence cytochrome P450 enzyme changes by altering transcriptional regulation of cytochrome P450 enzymes (Velenosi et al, 2014); modulating inflammation or parathyroid hormone (Michaud et al, 2006) or direct inhibition by uremic toxins (Sun et al, 2004; Barnes et al, 2014; Volpe et al, 2014).

The relationship between gut bacteria and host physiology/pathophysiology has been extensively studied. Alterations in gut microbiota composition have been linked with inflammatory bowel disease, obesity, cardiovascular disease, asthma, and cancer (Cho and Blaser, 2012; Lloyd-Price et al, 2016). Dysbiosis refers to changes in microbiota composition associated with a noninfectious disease state (Lloyd-Price et al, 2016). In a large cohort study of 1106 human stool samples, glomerular filtration rate was a major factor associated with altered microbiota composition (Falony et al, 2016). Thus, it comes as no surprise that patients with CKD also exhibit dysbiosis (Ren et al, 2020; Liu et al, 2021).

In this study, we hypothesized that CKD would cause uremia and gut microbiota changes detectable prior to downregulation of drug metabolizing enzymes. Bacterial alterations and metabolic changes have not been comprehensively studied with respect to altered drug metabolism throughout the temporal progression of CKD. Our study aims to characterize plasma and liver uremic toxins, the gut microbial composition and cytochrome P450 expression and activity over the progression of CKD.

## 2 Materials and methods

### 2.1 Animal model and study design

Sixty-seven male Wistar rats (6–8 weeks of age, ∼150 g) were obtained from Charles River Laboratories, Inc. After holding and acclimatization of 2 weeks experiments were initiated on the rats when they were 8 to 10 weeks of age and approximately 200 g (see Supplemental Table 3). The rats were randomized into 6 groups defined by time of euthanasia (day 0, 3, 7, 14, 28, and 42). Each time point consisted of 6 control and 6 CKD rats with the exception of the day 0 group that had only 6 rats and the day 42 CKD group that had 7 rats. Rats were housed with a same-group cage mate to minimize coprophagy alterations of the gut microbiota. We used the adenine model of CKD to allow evaluation of the progression of kidney disease (from normal through to severe CKD) on hepatic drug metabolism. Rats were given either 0.5% adenine supplemented chow to induce CKD or standard chow pair fed to match caloric intake. Rats were euthanized by isoflurane anesthetization followed by decapitation. Blood was collected in heparinized tubes and liver was snap-frozen in liquid nitrogen. Cecal samples were obtained on a sterile, single culture swab (BD) touched to an open incision of the caecum. All samples were stored at −80 °C until further analysis excluding the right kidneys, which were stored in 10% formalin. Animal studies were carried out in accordance with the Guide for the Care and Use of Laboratory Animals as per the US National Institutes of Health and approved by the University of Western Ontario Animal Care Committee.

### 2.2 Disease markers and histology

Conventional CKD markers urea and creatinine were measured in rat plasma and serum, respectively using standard methods by the Pathology and Laboratory Medicine group (www.lhsc.on.ca/palm/). Kidney tissue and histologic images were prepared as previously described (Feere et al, 2015). Light microscopy and photographs of prepared hematoxylin and eosin stained slides were obtained on a Leica DM1000 light microscope paired with a Leica DFC295 camera and Leica Application Suite v3.8.0 software.

### 2.3 Real-time polymerase chain reaction

Total mRNA was extracted from rat liver, tested for purity and quantified using quantitative polymerase chain reaction (PCR) with methods and validated primers as stated previously (Velenosi et al, 2014). Gene expression was normalized to *β*-actin using the ΔΔCT method.

### 2.4 Western blotting

Hepatic microsomal fractions were prepared by differential centrifugation as previously described (Feere et al, 2015). Western blot analysis was performed as previously described (Feere et al, 2015) and all blots were completed in duplicate for both Cyp3a2 and Cyp2c11. Antibodies for Cyp3a2 and Cyp2c11 were from Millipore and specificity was confirmed in one of our previous manuscripts (Velenosi et al, 2012).

### 2.5 Enzymatic activity

Enzymatic activity was analyzed by incubating microsomal fractions with testosterone, a known substrate of Cyp3A2 and Cyp2c11. Testosterone metabolites 6*β*OH-testosterone (Cyp3a2) and 16*α*OH-testosterone (Cyp2c11) were measured via mass spectrometry (MS) in a 96-well plate assay adapted from Feere et al (2015). In a final volume of 75 *μ*L, 0.2 mg/mL microsomal protein and reaction buffer (50 mM potassium phosphate with 2 mM MgCl_2_ pH 7.4) was incubated with 1 *μ*L of testosterone (Steraloids Inc) at concentrations of 12.5, 25, 75, 200, and 400 *μ*M for 10 minutes at 37 °C. All reactions were initiated with the addition of 1 mM NADPH (Sigma Aldrich) and shaken at 37 °C for 20 minutes before the reaction was terminated using 225 *μ*L ice-cold acetonitrile with 80 ng/mL flurazepam internal standard (Cerilliant). Plates were shaken, centrifuged at 4000 × *g* for 10 minutes, supernatant diluted 5-fold with Milli-Q water. Enzymatic products were separated on a Phenomenex Kinetex phenyl-hexyl column (1.7 *μ*m particle size, 50 mm × 2.1 mm) maintained at 40 °C in a Waters ACQUITY UPLC I-Class System. Mobile phase flow was set to 0.5 mL/min and consisted of ultra-performance liquid chromatography (UPLC)-grade water (A) and acetonitrile (B) both containing 0.1% formic acid with a gradient as follows: 0–0.5 minutes, 25% B; 0.5–2 minutes, 25%–35% B; 2–2.5 minutes, 35%–80% B; 2.5–3.5 minutes held at 80% B; and 3.5 minutes, 25% B. Analytes were detected using quadrupole time-of-flight MS on a Waters Xevo G2S quadrupole time-of-flight mass spectrometer and Waters ACQUITY I-Class UPLC with parameters as previously described (Feere et al, 2015). Mass-to-charge ratios for hydroxy testosterone were targeted (*m/z* = 305.2117) for quantification using QuanLynx v4.1 software. Michaelis-Menten curves were generated with GraphPad Prism (v6.0; GraphPad Software Inc).

### 2.6 Untargeted metabolomics

#### 2.6.1 Sample and batch preparation

Plasma and liver samples were prepared as previously described (Velenosi et al, 2016) with 3:1 ice-cold acetonitrile and 2.5 *μ*M chlorpropamide internal standard (Sigma Aldrich) then run on both the Waters ACQUITY UPLC HSS T3 (1.8 *μ*m particle size, 100 mm × 2.1 mm) reverse-phase liquid chromatography (RPLC) column and the Waters ACQUITY BEH Amide (1.7 *μ*m particle size, 100 mm × 2.1 mm) hydrophilic interaction liquid chromatography (HILIC) column in order to maximize coverage of polar and nonpolar metabolites. Supernatant was either diluted 5-fold in water for RPLC or directly injected for HILIC. Sample injection order was randomized, and a quality control sample made from pooled samples was run every 10 injections. All samples were run in a single batch for each biological matrix and column.

#### 2.6.2 Chromatography and MS

Columns were maintained at 45 °C and mobile phase flow set to 0.45 mL/min consisting of UPLC-grade water (A) and acetonitrile (B), both containing 0.1% formic acid. The RPLC analysis was run as previously described (Velenosi et al, 2016). The HILIC column followed a gradient of 0–0.5 minutes, 99% B; 0.5–6 minutes, 99%–50% B; 6–8 minutes, 50%–30% B; and 8–8.5 minutes, 30%–99% B. Samples were run separately in succession for both positive and negative electrospray ionization modes on the UPLC-QTof/MS instrument. Mass spectrometer source, method, calibration and other parameters were identical to those in Velenosi et al (2016) and data was collected by MassLynx v4.1 software (Waters).

#### 2.6.3 Data processing

Data processing for each run and ionization mode was performed separately in R studio (v3.2.3). MassLynx data files were converted to mzData files using convert.waters.raw package v1.0 (github.com/stanstrup/convert.waters.raw). Pooled samples were used to find the optimal peak picking parameters, retention time corrections and grouping parameters with the isotopologue parameter optimization package v1.0.0 (github.com/rietho/IPO/blob/master/vignettes/IPO.Rmd). The resulting parameters were inputted into the XCMS package v1.50.1 to pick appropriate peaks, integrate the area under the curve and replace zero values (Smith et al, 2006). The CAMERA package v1.32.0 was used to annotate possible isotopes and adducts (Kuhl et al, 2012). XCMS and CAMERA packages were used to combine positive and negative ionization modes together before normalizing to internal standard and applying a threshold of 30% variability of the quality control. Positive and negative modes were combined to make 1 dataset ready for statistical analysis with masses identified in both modes retained in the mode with greater intensity.

#### 2.6.4 Metabolite identification

The accurate monoisotopic mass (*m/z*) and fragmentation spectrum of each metabolite was used to identify metabolites via METLIN, MassBank, or Human Metabolome Database (Wishart et al, 2013). Metabolites were identified in accordance with the reporting standards for metabolite identification (Sumner et al, 2007) and analytical standards purchased for 9 compounds provided the highest level 1 metabolite identification.

### 2.7 In vitro assessment of uremic toxins on CYP3A4 expression

Human hepatoma Huh7 cells were maintained in Dulbecco’s modified Eagle’s medium supplemented with 10% FBS, 100 IU/mL penicillin, and 100 *μ*g/mL streptomycin and 2 mM l-glutamine. Cells were grown at confluence for 4 weeks prior to treatment to ensure adequate CYP3A4 expression levels (Sivertsson et al, 2010). Huh7 cells were treated with select uremic toxins: creatinine (2121.6 *μ*M), *p*-cresyl sulfate (186.1 *μ*M), indoxyl sulfate (1113.2 *μ*M), and urea (76.6 mM) for 24 hours. An indoxyl sulfate concentration-response effect on CYP3A4 mRNA expression was generated using a concentration range of indoxyl sulfate found in normal and patients with CKD (0–1000 *μ*M) with 40 g/L human serum albumin (HSA, Lee Biosolutions). Indoxyl sulfate was incubated in media containing 40 g/L HSA for 3 hours at 37 °C to allow for plasma protein binding equilibration prior to cell treatment. Cell viability was assessed using the 3-(4,5-dimethylthiazol-2-yl)-2,5-diphenyltetrazolium bromide assay as previously described (Arp et al, 2005).

### 2.8 Gut microbial sequencing

#### 2.8.1 Illumina sequencing

DNA was extracted from caecum swabs using the PowerSoil-96 Well DNA isolation kit from Qiagen using convenience modifications of the Earth Microbiome Project protocol (Gilbert et al, 2014). 288 unique primer combinations were established using the 515F and 806R barcoded primers (Caporaso et al, 2011; Gilbert et al, 2014) to amplify the V4 variable region of the 16S ribosomal RNA gene. Primers followed the template: Forward primer [5′-ACACTCTTT CCCTACACGACGCTCTTCCGATCTnnnn (8)GTGCCAGCMGCCGCGGTAA-3′] and reverse primer [5′-CGGTCTCGGCATTCCTGCTGAACGCTCTTCCGATCTnnnn (8)GGACTACHVGGGTWTCTAAT-3′] where the 5′-end is the Illumina adaptor sequence, the nnnn indicates 4 random nucleotides, (8) represents 1 of 36 barcoded sequences and the 3′-end is the primer region for V4 (Supplemental Table 1). Amplification was carried out in 42 *μ*L of total volume with 20 *μ*L of primer mix (3.2 pmol/*μ*L per primer), 20 *μ*L of GoTaq Hot Start Mastermix (Thermo Scientific) and 2 *μ*L of template DNA then run for 2 minutes at 95 °C followed by 25 cycles of 1 minute at 95 °C; 1 minute at 52 °C and 1 minute at 72 °C excluding a final elongation. Barcoded PCR products were quantified with a Qubit double-stranded DNA assay kit on a Qubit 2.0 (Life Technologies), normalized by amount of DNA, pooled then purified with a PCR clean-up column. The cleaned DNA was amplified once more with primers OLJ139 [5ʹAATGATACGGCGACCACCGAGATCTACACTCTTTCCCTACACGA3ʹ] and OLJ140 [5ʹCAAGCAGAAGACGGCATACGAGATCGGTCTCGGCATTCCTGCTG AAC3ʹ] before paired-end sequencing on the Illumina MiSeq platform at the London Regional Genomics Centre (lrgc.ca).

#### 2.8.2 Data processing

Paired reads, each 220 bp long, were demultiplexed using custom scripts and raw reads were overlapped with a minimum 30 nucleotides using Pandaseq (v2.5) (Masella et al, 2012) then filtered with in-house Perl and UNIX scripts to ensure exact barcode matching and primer matching with up to 2 allowable mismatches (Gloor et al, 2010). Operational taxonomic units (OTUs) were clustered at 97% identity using the uSearch (v7.0.1090) tool (Edgar, 2010) and the most abundant sequence in the OTU was annotated via the mothur script (Schloss et al, 2009) to search the Silva 16S ribosomal RNA gene reference database (Silva.nr_v119) (Quast et al, 2013; Yilmaz et al, 2014). In mothur, a bootstrap cut-off of 70% was used for taxonomical identification and redundancy. A total of 1199 OTUs were retained across all samples (Supplemental Table 2). In R studio (v3.2.3) the zCompositions (v1.0.3-1) package (Martín-Fernández et al, 2003) was used for zero-replacement before data was centered-log ratio transformed for compatibility with downstream multivariate statistical analysis (Gloor and Reid, 2016; Gloor et al, 2016a).

### 2.9 Statistical analysis

#### 2.9.1 Disease markers, real-time PCR, western blotting, and enzymatic activity assay

Cytochrome P450 measurements and disease markers urea and creatinine are presented as mean ± SEM and analyzed by two-way ANOVA paired with Sidak’s multiple comparisons test. ∗*P* < .05 compared with matching day control indicates significance.

#### 2.9.2 Untargeted metabolomics

MassLynx software and the EZInfo v2.0 package (Umetrics) were used to perform principal component analysis (PCA) to evaluate the initial separation between CKD and control over time for each of the 4 analytical runs. Data was Pareto scaled and pooled samples were run to confirm minimal variance. Multivariate analysis was performed on each day of each run. EZInfo was used to generate orthogonal partial least squares discriminant analysis (OPLS-DA) of the original PCA (Supplemental Fig. 1). To assess multivariate OPLS-DA sufficiency, each comparison received a goodness of fit value ratio threshold (*R*^*2*^/*Q*^*2*^ < 2) (Triba et al, 2015). Subsequent thresholds were applied (variable importance in projection > 0.8; *P* (corr)[1] > .4 or < −.4) by finding the variable importance in projection and the *P* (corr)[1] axis as a measure of magnitude and difference between treatments (Farrés et al, 2015) (Supplemental Fig. 1). Only metabolites that met or exceeded the thresholds on ≥2 consecutive time points were retained for comparison with univariate and correlative analyses. Univariate analysis was performed by the open-access online software MetaboAnalyst 3.0 to conduct a *P* value corrected (false discovery rate, 0.05) independent two-way ANOVA on each metabolite via the “Time series” and “Two-factor independent samples” applications (Wishart et al, 2013). Significance (*P* < .05) was required for both “Time” and “Disease” to retain the metabolite for comparison with multivariate and correlative analyses. Spearman correlations were conducted between each metabolite and the mRNA, protein or enzymatic activity levels of each enzyme. Metabolomics datasets were matched by sample to the corresponding cytochrome P450 dataset and correlation coefficients (*r* values) manually filtered with high stringency (*r* > 0.65 or *r* < −0.65). Metabolites that did not also satisfy univariate analysis were removed from the correlation subset. Metabolites that did not satisfy multivariate analysis are indicated but retained to capture biologically relevant changes independent of magnitude.

#### 2.9.3 Cecal microbiota

Multivariate PCA was performed in EZInfo as described above for untargeted metabolomics excluding scaling. To evaluate univariate differences between CKD and control groups, the effect size and overlap for each bacterial taxonomic group was calculated for each time point individually using the R package ALDEx2 (v1.2.0) (bioconductor.org/packages/release/bioc/html/ALDEx2.html) (Fernandes et al, 2014; Gloor et al, 2016a). Severe thresholds were applied to both effect size (>1.5 or <−1.5) and overlap (<6.5%) for each bacterial abundance (Macklaim et al, 2013; Gloor et al, 2016b). Significance was defined as satisfying the effect size and overlap thresholds with 95% confidence. Species and strain information were manually searched using the Targeted Loci Nucleotide BLAST application through National Center for Biotechnology Information (blast.ncbi.nlm.nih.gov/Blast.cgi).

## 3 Results

### 3.1 Model of CKD progression

**Fig. 1:**
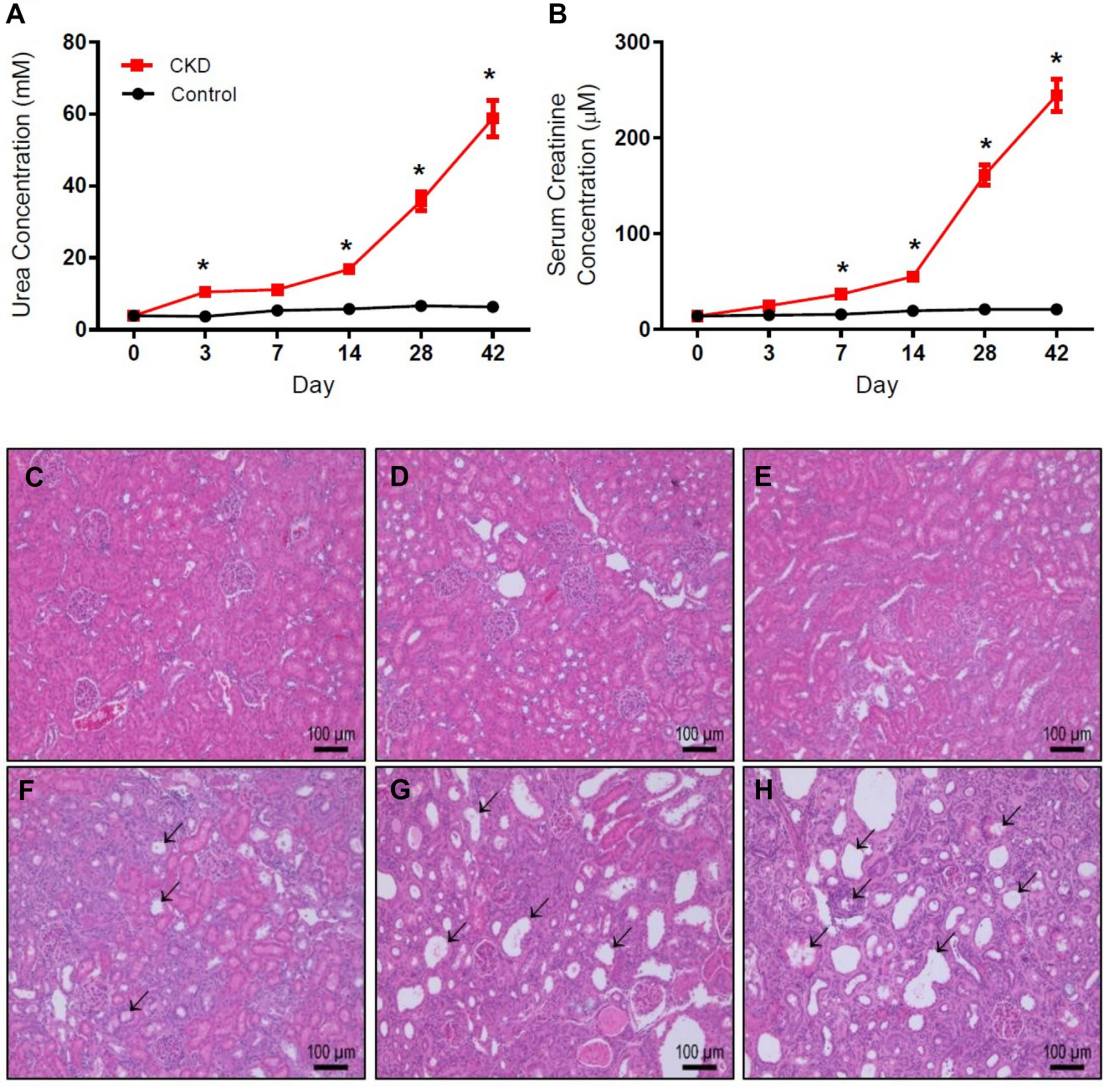
Assessment of CKD in Wistar rats orally administered 0.5% adenine over 42 days. (A) Plasma urea (mM) and (B) serum creatinine (*μ*M) concentrations of control and CKD rats presented as mean ± SEM. ∗*P* < .05 when compared with matching day control; *n* ≥ 6. H&E stained rat kidney sections from day 0 control (C) and CKD days 3 (D), 7 (E), 14 (F), 28 (G), and 42 (H). Arrows indicate enlarged nephron tubules and areas of fluid retention. Inflammation and atrophy are evident on days 14, 28, and 42.

CKD markers urea and creatinine both showed significant increase in CKD rat plasma by day 3 (urea) and day 7 (creatinine) and both were significantly elevated by day 14 (Fig. 1, A and B). This early rise in known kidney function biomarkers demonstrates kidney function was impacted very early in the adenine model. This increase continued to a 9-fold and 11-fold difference between CKD and control for urea and creatinine, respectively, on day 42. Kidney histology showed enlarged tubules, inflammation and fibrosis by day 14 through to day 42 (Fig. 1, C–H). Animal weights did not change between groups (Supplemental Table 3).

### 3.2 Hepatic Cyp3a2 and Cyp2c11 gene expression, protein expression, and activity over CKD progression

**Fig. 2:**
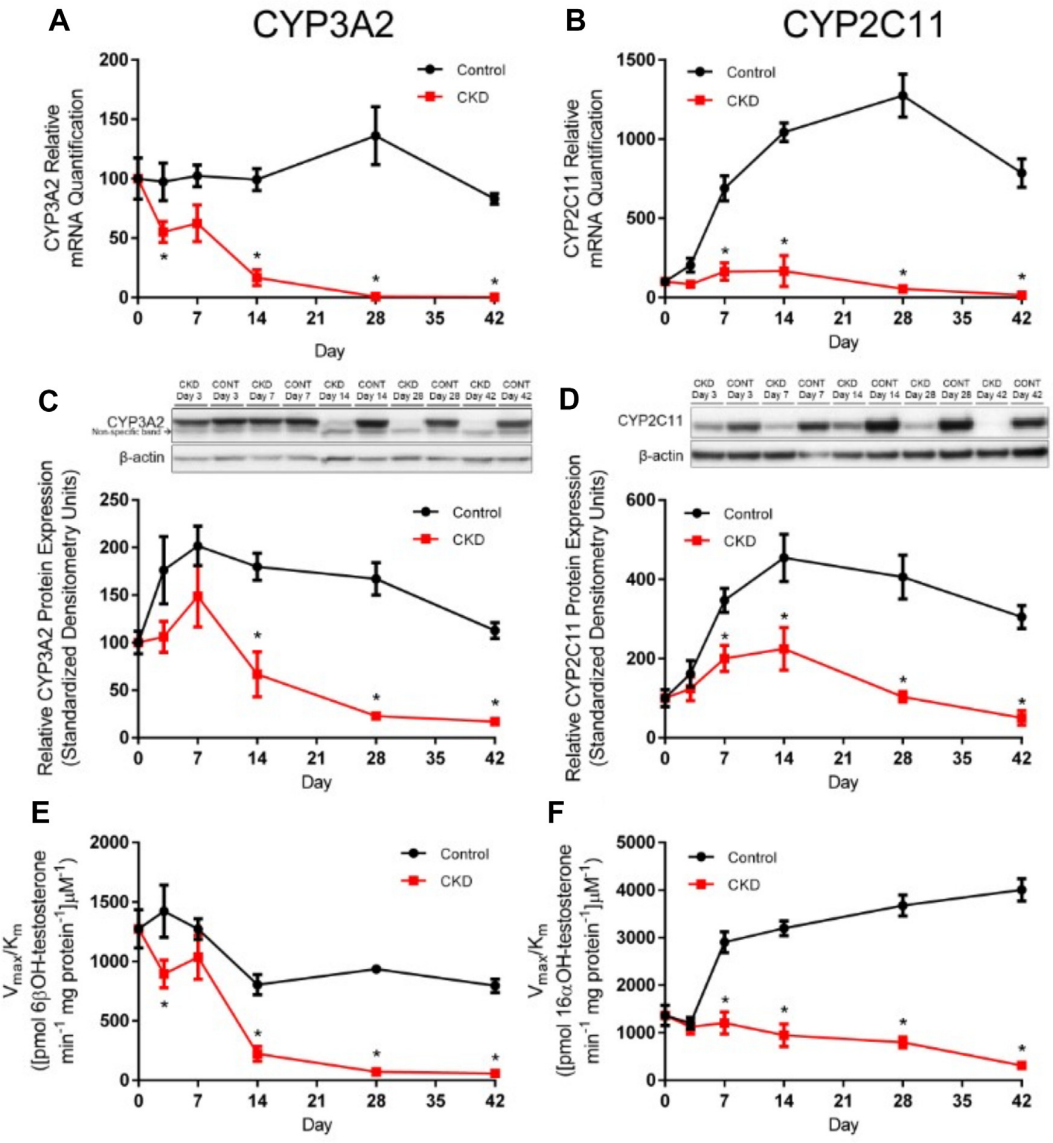
Relative mRNA expression, protein expression, and enzymatic activity levels of CYP3A2 and CYP2C11. CYP3A2 (A) and CYP2C11 (B) mRNA expression and protein expression, CYP3A2 (C) and CYP2C11 (D), with representative western blots. Values were relative to *β*-actin represented as the mean ± SEM, normalized to control day 0 and arbitrarily defined as 100%. Enzymatic activity of CYP3A2 (E) and CYP2C11 (F) in control and CKD rats represented as the mean intrinsic clearance V_max_/K_m_ [(mL/min/mg protein)] of testosterone metabolite ± SEM. ∗*P* < .05 when compared with matching day control; *n* ≥ 6.

Cyp3a2 mRNA expression was minimally decreased on day 3, recovered on day 7, then declined substantially by day 14 (−83%, *P* < .001), a decrease that persisted to day 42 (−99%, *P* < .001) (Fig. 2A). Cyp2c11 mRNA expression was unchanged on day 3 but largely increased in the control group on day 7 leaving CKD rats well below normal (−76%, *P* < .001) (Fig. 2B). On day 14 (−84%, *P* < .001), day 28 (−96%, *P* < .001), and day 42 (−98%, *P* < .001) the CKD Cyp2c11 mRNA expression was decreased in comparison to control.

Significant decreases in Cyp3a2 protein expression were observed in CKD animals on day 14 (−63%, *P* < .001), day 28 (−86%, *P* < .001), and day 42 (−85%, *P* < .01) (Fig. 2C). Cyp2c11 protein quantification also shows depletion in CKD but starting on day 7 (−42%, *P* < .05) through to day 42 (−83%, *P* < .001) (Fig. 2D).

Cyp3a2 intrinsic activity in CKD rats decreased on day 3, recovered on day 7 and fell again on day 14 (−72%), day 28 (−92%), and day 42 (−93%) compared with control (Fig. 2E). The intrinsic activity of Cyp2c11 was lower in CKD compared with control as early as day 7 (−59%) and day 42 was 92% lower than control (Fig. 2F). Michaelis-Menten parameters are summarized (Table 1).

**Table 1:** CYP3A2 and CYP2C11 enzymatic activity over CKD progression 6*β*OH-testosterone (CYP3A2) and 16*α*OH-testosterone (CYP2C11) production of liver microsomes measured after incubation with NADPH and testosterone. V_max_ values are in pmol/min/mg protein and Km values are *μ*M.

	V_max_		K_m_		Intrinsic Clearance (V_max_/K_m_)	
	Control	CKD	Control	CKD	Control	CKD
**6*β*OH-testosterone (CYP3A2)**						

Day 0	35,938 ± 2841	35,938 ± 2841	29.5 ± 9.02	29.5 ± 9.02	1275 ± 393.87	1275 ± 393.87
Day 3	56,184 ± 5640	26,425 ± 2097∗	40.1 ± 14.46	29.8 ± 9.13	1422 ± 540.42	894 ± 288.25∗
Day 7	49,912 ± 1806	42,396 ± 4911	40.2 ± 5.218	42.7 ± 17.48	1272 ± 214.04	1036 ± 489.05
Day 14	38,223 ± 2797	12,804 ± 1983∗	49.4 ± 12.34	59.4 ± 29.88	804 ± 207.09	222 ± 152.72∗
Day 28	61,702 ± 3069	10,102 ± 1289∗	66.6 ± 10.43	141.6 ± 44.74∗	936 ± 86.36	71 ± 21.50∗
Day 42	45,706 ± 2266	7849 ± 1297∗	58.5 ± 9.46	135.6 ± 56.32∗	795 ± 139.12	58 ± 24.76∗

**16*α*OH-testosterone (CYP2C11)**						

Day 0	17,983 ± 1210	17,983 ± 1210	14.2 ± 4.56	14.2 ± 4.56	1365 ± 518.05	1365 ± 518.05
Day 3	25,892 ± 2353	15,453 ± 1195	21.8 ± 8.28	13.8 ± 5.13	1202 ± 298.73	1123 ± 357.65
Day 7	41,420 ± 2488	27,114 ± 3431	14.3 ± 4.09	23.5 ± 12.17	2907 ± 541.70	1206 ± 607.94∗
Day 14	51,824 ± 2556	17,521 ± 2406∗	16.9 ± 3.75	18.6 ± 11.18	3199 ± 377.15	948 ± 523.91∗
Day 28	72,358 ± 2064	16,236 ± 1548∗	20.1 ± 2.45	21.2 ± 8.52	3679 ± 548.84	797 ± 273.19∗
Day 42	62,571 ± 3147	6658 ± 513∗	16.2 ± 3.71	22.6 ± 7.21	4005 ± 570.00	312 ± 179.33∗

CKD, chronic kidney disease.

∗*P* < .05 compared with matching day control; *n* ≥ 6.

### 3.3 Plasma and liver metabolomics

**Fig. 3:**
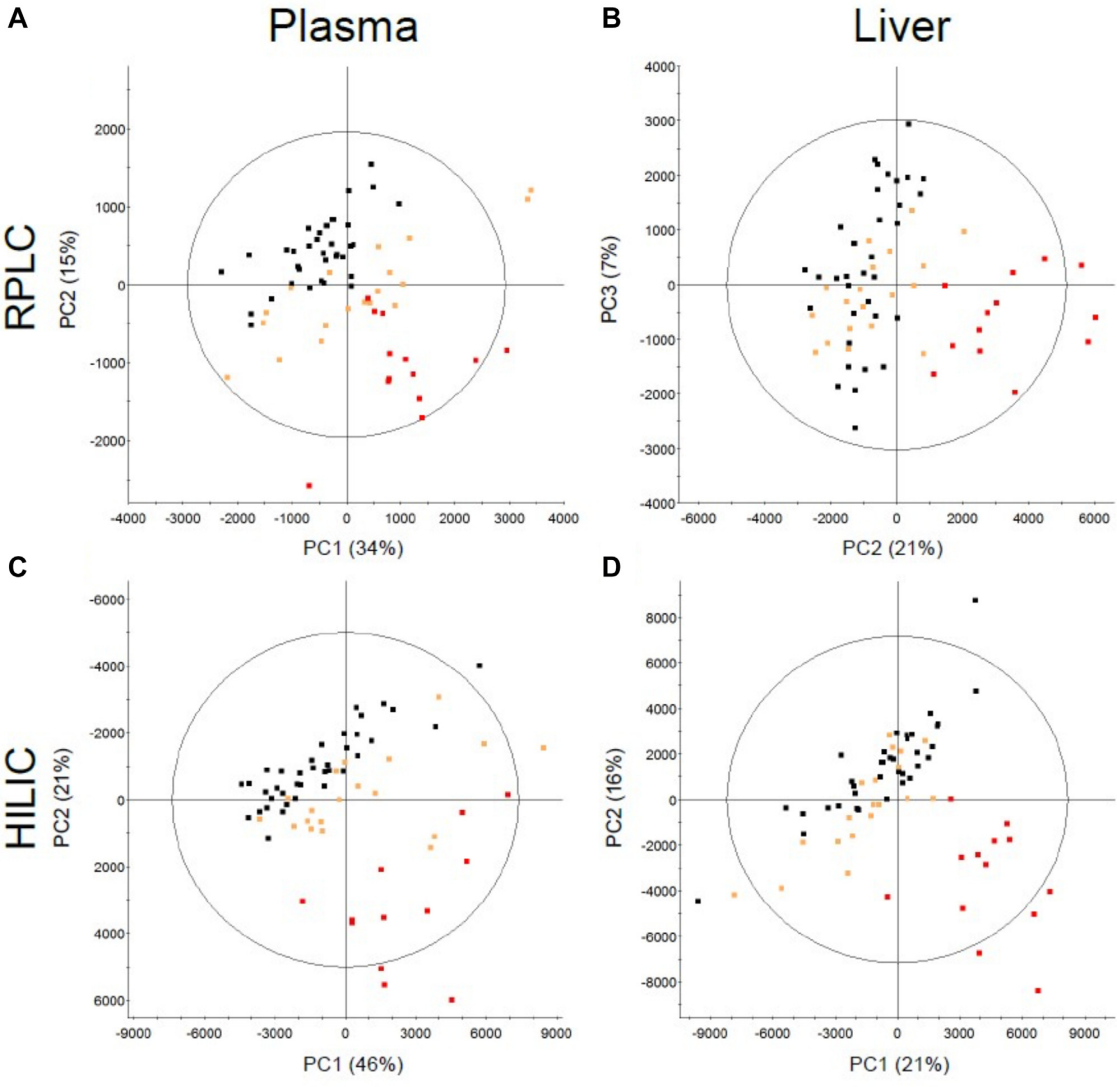
Unsupervised PCA plots of rat plasma (A) and liver (B) metabolome separated by RPLC. PCA of plasma (C) and liver (D) metabolome separated by HILIC. Each point is either control (■), early stage CKD defined by day 3, 7, and 14 (), or late stage CKD defined by days 28 and 42 (). Each axis is either the first [1], second [2] or third [3] principal component (PC) showing the 2 components representing the largest variation between groups. Placement of each sample is determined by the metabolite composition within each sample and clustered samples share similar compositions. Data are centered and Pareto-scaled. Select rat samples were removed as outliers (A) no outliers, (B) a day 28 CKD sample, (C) a day 3 and day 42 control, and (D) a day 7 control sample.

Untargeted metabolomics analysis was used to assess changes in metabolite composition. PCA clearly separated CKD and control for both rat plasma and liver samples (Fig. 3). Early disease stages are arbitrarily defined as days 3 and 14 and late stages days 28 and 42. *R*^*2*^ and *Q*^*2*^ parameters were used to accompany the interpretation of OPLS-DA plots (Table 2). Metabolites in rat plasma were well separated from control as early as day 3 when using RPLC (Fig. 3A). Liver RPLC showed far less separation between control and CKD before day 28 (Fig. 3B). The HILIC column showed separation back to day 7 except for poor *Q*^*2*^ values on day 14 in both plasma and liver samples (Fig. 3, C and D).

**Table 2:** Multivariate OPLS-DA parameters *R*^*2*^ and *Q*^*2*^ *R*^*2*^ and *Q*^*2*^ values for plasma and liver metabolomics using RPLC and HILIC across all time points.

	RPLC				HILIC			
**Rat plasma**								

Day 42	Comp no.	*R*^*2*^	*Q*^*2*^	*R*^*2*^/*Q*^*2*^	Comp no.	*R*^*2*^	*Q*^*2*^	*R*^*2*^/*Q*^*2*^
	[1]	0.9859	0.9175	1.0745	[1]	0.9821	0.5936	1.6546
	[2]	0.9859	0.9493	1.0385	[2]	0.9821	0.8064	1.2180
					[3]	0.9821	0.8677	1.1319
Day 28	Comp no.	*R*^*2*^	*Q*^*2*^	*R*^*2*^/*Q*^*2*^	Comp no.	*R*^*2*^	*Q*^*2*^	*R*^*2*^/*Q*^*2*^
	[1]	0.9982	0.6410	1.5572	[1]	0.9734	0.8402	1.1586
	[2]	0.9982	0.8176	1.2210	[2]	0.9734	0.9491	1.0255
							
Day 14	Comp no.	*R*^*2*^	*Q*^*2*^	*R*^*2*^/*Q*^*2*^	Comp no.	*R*^*2*^	*Q*^*2*^	*R*^*2*^/*Q*^*2*^
	[1]	0.8940	0.5438	1.6439	[1]	0.7248	0.2872	2.5240
	[2]	0.8940	0.5203	1.7181	[2]	0.7248	0.5121	1.4152
							
Day 7	Comp no.	*R*^*2*^	*Q*^*2*^	*R*^*2*^/*Q*^*2*^	Comp no.	*R*^*2*^	*Q*^*2*^	*R*^*2*^/*Q*^*2*^
	[1]	0.9628	0.5418	1.7770	[1]	0.9169	0.5979	1.5336
	[2]	0.9628	0.7150	1.3466	[2]	0.9169	0.8431	1.0876
							
Day 3	Comp no.	*R*^*2*^	*Q*^*2*^	*R*^*2*^/*Q*^*2*^	Comp no.	*R*^*2*^	*Q*^*2*^	*R*^*2*^/*Q*^*2*^
	[1]	0.8680	0.5969	1.4542	[1]	0.9873	0.2812	3.5112
	[2]	0.8680	0.5420	1.6016	[2]	0.9873	0.6354	1.5538
					[3]	0.9873	0.7510	1.3146
**Rat liver**								

Day 42	Comp no.	*R*^*2*^	*Q*^*2*^	*R*^*2*^/*Q*^*2*^	Comp no.	*R*^*2*^	*Q*^*2*^	*R*^*2*^/*Q*^*2*^
	[1]	0.9753	0.7750	1.2585	[1]	0.9832	0.8722	1.1273
	[2]	0.9753	0.8708	1.1200	[2]	0.9832	0.9194	1.0694
	[3]	0.9753	0.9214	1.0585				
Day 28	Comp no.	*R*^*2*^	*Q*^*2*^	*R*^*2*^/*Q*^*2*^	Comp no.	*R*^*2*^	*Q*^*2*^	*R*^*2*^/*Q*^*2*^
	[1]	0.9691	0.7830	1.2377	[1]	0.9900	0.9034	1.0958
	[2]	0.9691	0.9284	1.0438	[2]	0.9900	0.9286	1.0661
							
Day 14	Comp no.	*R*^*2*^	*Q*^*2*^	*R*^*2*^/*Q*^*2*^	Comp no.	*R*^*2*^	*Q*^*2*^	*R*^*2*^/*Q*^*2*^
	[1]	0.8357	0.0070	118.877	[1]	0.9712	0.4582	2.1194
	[2]	0.8357	0.2953	2.8298	[2]	0.9712	0.6679	1.4541
					[3]	0.9712	0.7677	1.2651
Day 7	Comp no.	*R*^*2*^	*Q*^*2*^	*R*^*2*^/*Q*^*2*^	Comp no.	*R*^*2*^	*Q*^*2*^	*R*^*2*^/*Q*^*2*^
	[1]	0.9973	0.3534	2.8218	[1]	0.9512	0.5142	1.8498
	[2]	0.9973	0.7233	1.3787	[2]	0.9512	0.7394	1.2864
	[3]	0.9973	0.8425	1.1837				
Day 3	Comp no.	*R*^*2*^	*Q*^*2*^	*R*^*2*^/*Q*^*2*^	Comp no.	*R*^*2*^	*Q*^*2*^	*R*^*2*^/*Q*^*2*^
	[1]	0.8938	0.5384	1.6601	[1]	0.9512	0.5142	1.8498
	[2]	0.8938	0.5749	1.5548	[2]	0.9512	0.7394	1.2864
					[3]	0.9873	0.7510	1.3146

### 3.4 Cytochrome P450 enzymes and uremic toxins

**Fig. 4:**
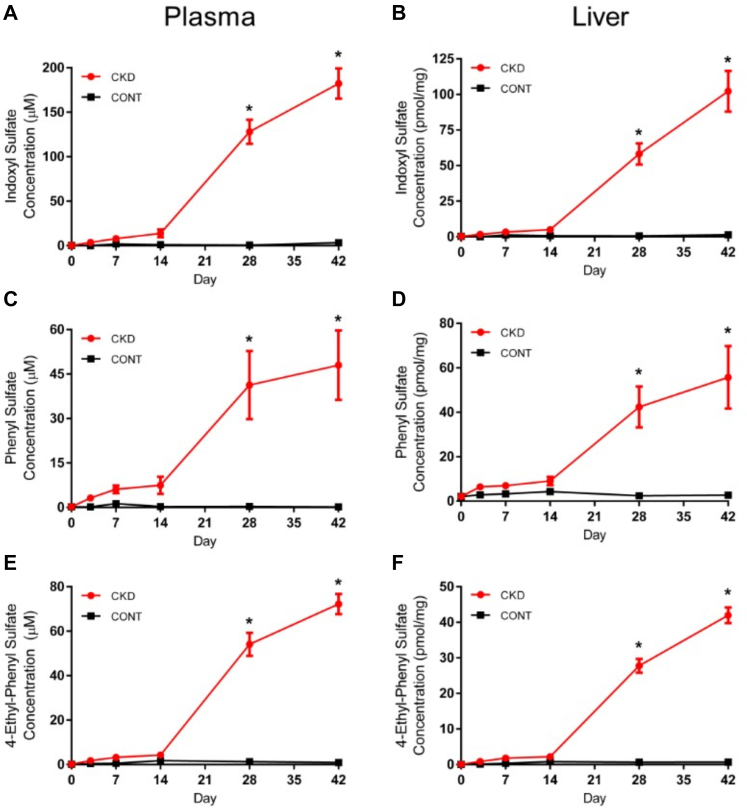
Quantitative analysis of metabolites indoxyl sulfate, phenyl sulfate, and 4-ethylphenyl Sulfate. Plasma indoxyl sulfate (A), phenyl sulfate (C), 4-ethylphenyl sulfate (E) (*μ*M) and liver indoxyl sulfate (B), phenyl sulfate (D) and 4-ethylphenyl sulfate (F) (pmol/mg liver tissue) concentrations obtained via untargeted metabolomics. Results are presented as mean ± SEM, ∗*P* < .0001 when compared with same day control; *n* ≥ 6.

After satisfying multivariate and univariate analysis, metabolites were correlated to cytochrome P450 mRNA, protein and enzymatic activity. There were 204 unique *m/z* ratios identified across all 4 runs that correlated with either Cyp3a2 or Cyp2c11 (Supplemental Table 4). Of these 204 *m/z* ratios, 9 metabolites were identified at identification level 1 using purchased standards. These metabolites include: allantoin, l-carnitine, creatinine, 2,8-dihydroxyadenine, equol-4/7-*O*-glucuronide, 4-ethylphenyl sulfate, indoxyl sulfate, pantothenic acid (vitamin B5) and phenyl sulfate. Indoxyl sulfate, phenyl sulfate, and 4-ethylphenyl sulfate had increased concentration (*P* < .0001) on days 28 and 42 for both plasma and liver tissue (Fig. 4).

### 3.5 Indoxyl sulfate downregulates hepatic CYP3A4 expression in vitro

**Fig. 5:**
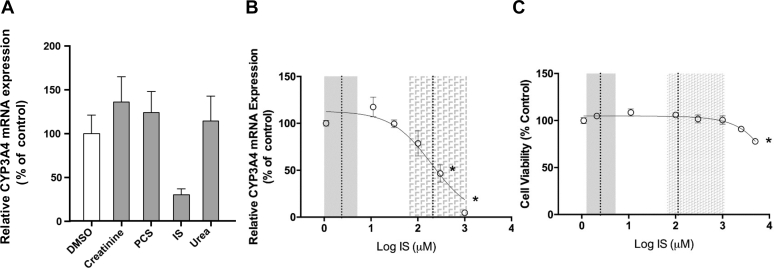
(A) The effect of select uremic toxins on CYP3A4 expression in Huh7 cells. (B) Concentration dependent effect of indoxyl sulfate (IS) on CYP3A4 expression in Huh7 cells. (C) Cell viability in the presence of increasing indoxyl sulfate concentrations. The gray solid area represent normal plasma indoxyl sulfate concentrations mean (dashed line) ± 2 SD. The gray-checkered area represents mean uremic concentration (dashed line) −2 SD to the highest individual measured uremic concentration. Results are represented as mean ± SEM, ∗*P* < .05; *n* ≥ 3. PCS, p-cresyl sulfate.

To investigate the mechanism of CYP3A downregulation in CKD, human hepatoma Huh7 cells were treated with selected uremic toxins. As an initial proof-of-concept screen, concentrations of uremic toxins used were the highest reported in patients with stage 5 CKD (Vanholder et al, 2003). A 24-hour indoxyl sulfate treatment decreased CYP3A4 mRNA expression by 70% in Huh7 cells (Fig. 5A). Other individual uremic toxins did not affect CYP3A4 mRNA expression. Indoxyl sulfate is a protein bound uremic toxin; therefore, the concentration dependence of this effect was evaluated in the presence of 40 g/L HSA in the culture medium. Treatment with indoxyl sulfate for 48 hours produced a concentration-dependent decrease in CYP3A4 mRNA expression as the concentration was increased from those measured in healthy controls to concentrations in patients with CKD (IC_50_ = 113.0 ± 3.5 *μ*M) (Fig. 5B). A significant decrease in the steady-state levels of CYP3A4 mRNA was demonstrated when Huh7 cells were treated with ≥300 *μ*M indoxyl sulfate in the presence of 40 g/L HSA supplemented media (Fig. 5B, *P* < .05). Huh7 cells treated with indoxyl sulfate in the uremic range resulted in a 21%–95% decrease in CYP3A4 mRNA expression. Cell viability was unaffected by indoxyl sulfate in media containing HSA after 48 hour treatment at clinically relevant concentrations (Fig. 5C).

### 3.6 Cecal microbiota

**Fig. 6:**
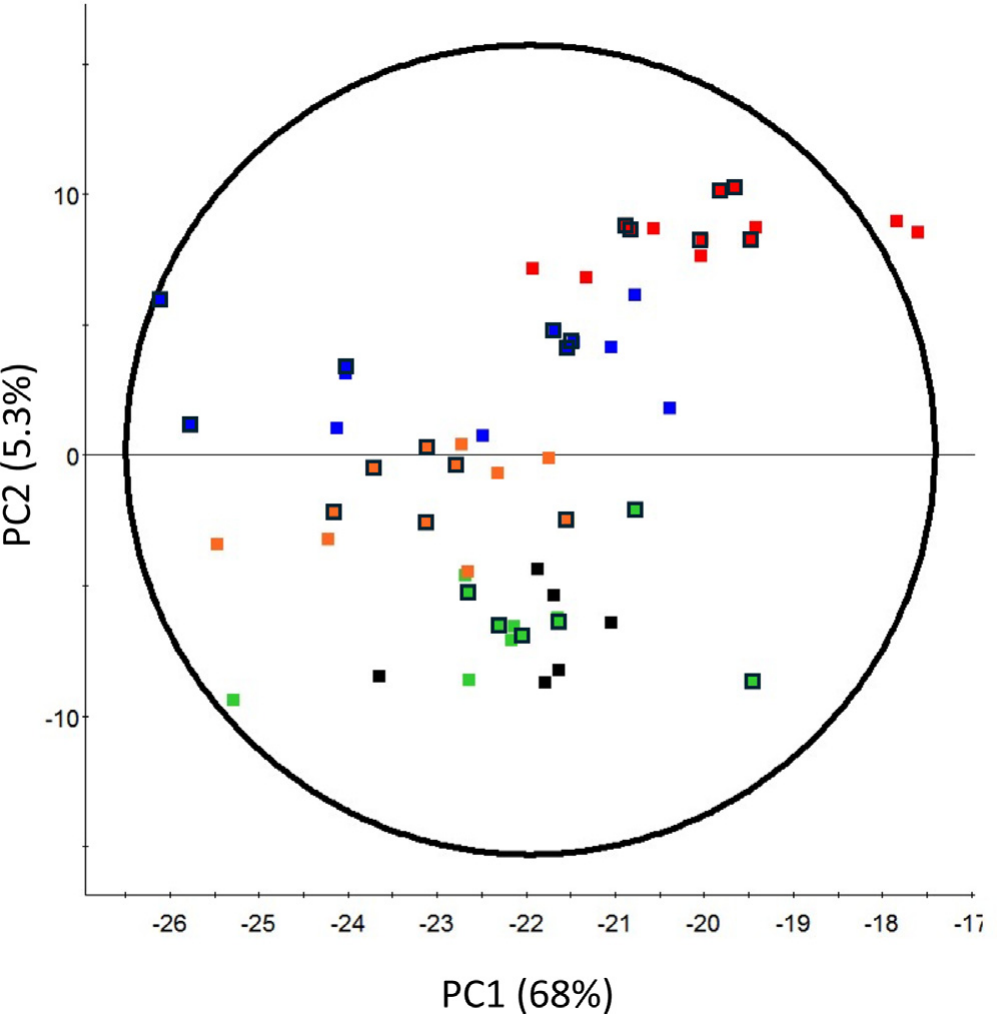
Unsupervised PCA of control and CKD rat caecum bacterial sequences colored by (day 0 (■) 3 (), 14 (), 28 (), and 42 (). Control samples are shown with a black box around the square data point, CKD samples do not have a black box around the data point. Data are centered without scaling. PC, principal component.

**Fig. 7:**
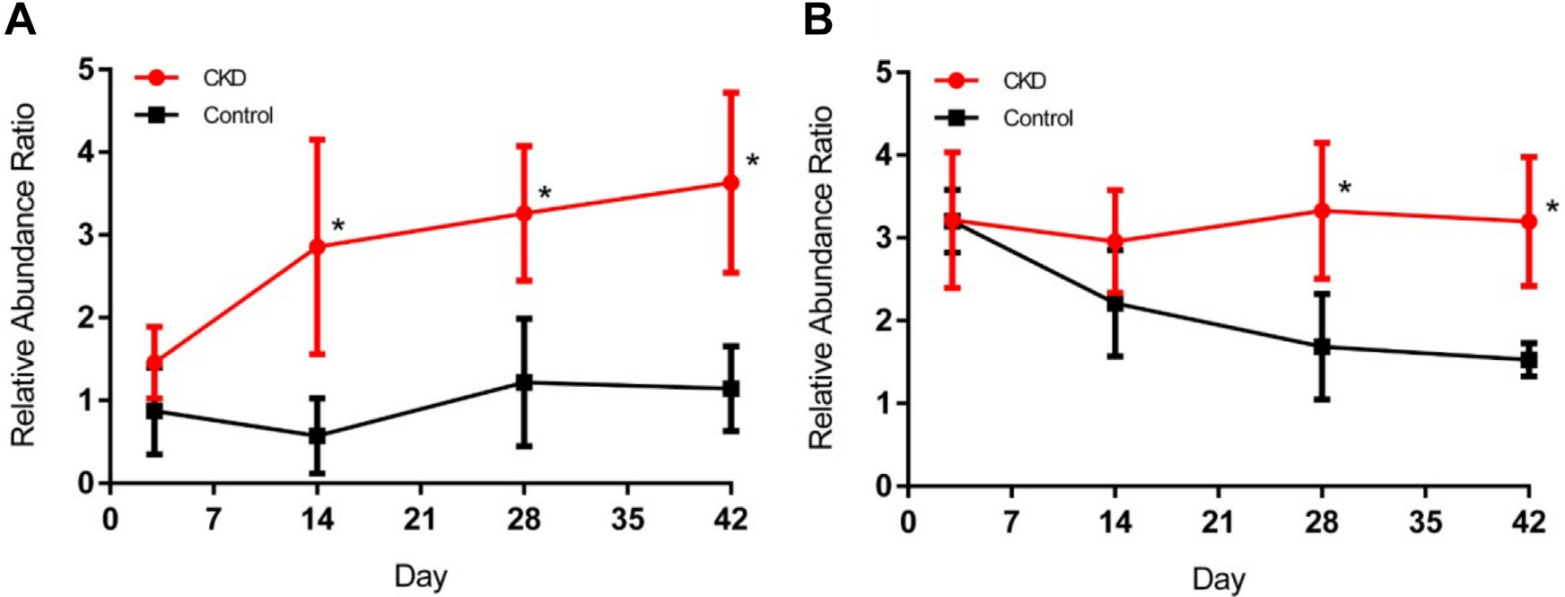
Mean relative abundance of genus *Turicibacter* (A) and genus *Parabacteroides* (B) displayed as the centered-log ratio-transformed values of the OTUs ± 95% confidence interval using R v3.2.3 package ALDEx2 v1.2.0. ∗Absolute effect size ≥ 1.5 and overlap < 6.5% compared with same day control; *n* ≥ 6.

To understand if the gut microbiota was changing in parallel with metabolite changes, next-generation Illumina sequencing was used to assess the bacterial composition of the caecum (Supplemental Table 2). Exploratory PCA ordination showed that the caecum samples had high intrinsic biological variation, but separated by time, regardless of disease state (Fig. 6A). A multivariate analysis found that CKD and control separated into distinct groups at day 28 and 42 (day 28: *R*^*2*^ = 0.97; *Q*^*2*^ =0.71 and day 42: *R*^*2*^ = 0.98; *Q*^*2*^ =0.70) (Fig. 6B). Effect size and overlap of each OTU relative abundance was tabulated and assessed for trends. Only 2 bacterial OTUs changed between control and CKD on ≥2 consecutive days with respect to effect size and overlap so these 2 OTUs were evaluated for time dependent changes between control and CKD. The first OTU was from the phylum Firmicutes and genus *Turicibacter* and was significantly higher in CKD rats compared with control animals on days 14, 28, and 42 (Fig. 7A) with an increasing trend associated with disease progression. The second OTU from phylum Bacteroidetes and genus *Parabacteroides* showed a significant decrease in control rats over time (Fig. 7B).

## 4 Discussion

The pharmacokinetics of many drugs are unpredictably altered in CKD, making these patients susceptible to adverse drug events. Hepatic cytochrome P450s play a crucial role in nonrenal drug clearance and alterations in these cytochrome P450s may contribute to pharmacokinetic changes observed in CKD. Cytochrome P450 downregulation has been associated with decreased renal clearance and consequent retention of uremic toxins in animal models of CKD. Mechanistic studies suggest the involvement of various pathways from pretranscriptional regulation to direct inhibition by uremic toxins, inflammatory factors, and hormones (Guévin et al, 2002; Sun et al, 2004; Michaud et al, 2006, 2008; Barnes et al, 2014; Velenosi et al, 2014; Volpe et al, 2014). Uremic toxins are also suggested to change the relative abundance of gut bacteria to favor uremic-toxin producing microbes and create a state of dysbiosis in patients with CKD (Felizardo et al, 2016). Changes in metabolite or toxin production as a consequence of the altered microbiota may exacerbate CKD progression and potentially cytochrome P450 downregulation. However, the pathophysiological factors of uremia and dysbiosis have yet to be evaluated temporally. In this manuscript, uremia and dysbiosis were characterized over CKD progression to identify potential mediators of cytochrome P450 downregulation.

Cyp3a2 and Cyp2c11 were both downregulated by CKD with respect to mRNA expression, protein expression, and enzymatic activity as previously observed (Velenosi et al, 2012). These findings suggest these cytochrome P450s were initially influenced at the transcriptional level, consequently leading to altered protein expression and enzyme activity. Expression of Cyp3a2 in control rats was stable throughout the study, whereas expression started to decrease on day 14 in CKD rats. These observations suggest the removal, inhibition, or downregulation of a constitutive factor required for expression, potentially mediated by the increase in uremic toxins (Wright et al, 1997). In contrast, control rats exhibited an increase in Cyp2c11 expression as early as day 7 but this increase was not evident in CKD rats. Increased Cyp2c11 expression over time has been described in healthy male juvenile rats, where it was suspected to reflect increased testosterone levels during puberty (Wright et al, 1997). Furthermore, CKD has been associated with hypogonadism and testosterone deficiency (Carrero et al, 2011). Cyp2c11 is also influenced by alterations in the normally cyclic levels of growth hormone (GH) where continuous GH release or loss of GH production will both downregulate Cyp2c11 (Kaufhold et al, 2002).

The differing trends between Cyp3a2 and Cyp2c11 may be attributed to nuclear receptor differences. Cyp2c11 is less dependent on hepatocyte nuclear factor 4*α* induction, and CKD-induced receptor binding inhibition is less extensive for Cyp2c11 than it is Cyp3a2 (Moazzen et al, 2014; Feere et al, 2015). Alternatively, it is also possible that removal or inhibition of shared nuclear receptor pregnane X receptor or reduced receptor binding of RNA polymerase II is affecting both enzymes but in different manners depending on substrate availability (Velenosi et al, 2014; Feere et al, 2015).

Plasma samples showed greater metabolomics separation in earlier stages of disease (days 3–14) than liver samples. This suggests CKD first inflicts a uremic environment in the plasma before infiltrating the liver. Uremic changes also overlapped with the early changes in Cyp3a2 and Cyp2c11, supporting the hypothesis that uremic toxins are involved with cytochrome P450 regulation, although this remains to be proved mechanistically. Metabolites from each metabolomics run were subjected to correlation analysis with Cyp3a2 or Cyp2c11 mRNA, protein or enzymatic activity levels. Of the 204 *m/z* features retrieved, 8 of the 9 identified at level 1 classification were increased with CKD progression [allantoin, creatinine, 2,8-dihydroxyadenine, pantothenic acid (vitamin B_5_), indoxyl sulfate, phenyl sulfate, equol-4/7-*O*-glucuronide, and 4-ethylphenyl sulfate]. l-carnitine was the only level 1 metabolite that showed a positive correlation with cytochrome P450 downregulation, decreasing over CKD progression.

• Gut derived uremic toxins that are potentially involved in cytochrome P450 downregulation from this study include indoxyl sulfate, phenyl sulfate, 4-ethylphenyl sulfate, equol-4/7-*O*-glucournide and products of L-carnitine metabolism. Indoxyl sulfate and phenyl sulfate are 2 highly retained gut-derived uremic toxins (Wikoff et al, 2011; Leong and Sirich, 2016) both found in patients with CKD and animal models (Zhao et al, 2013; Velenosi et al, 2016). Indoxyl sulfate and phenyl sulfate have been associated with altered drug metabolism both through transcriptional regulation (Guévin et al, 2002), and indoxyl sulfate as a direct inhibitor of cytochrome P450 activity (Volpe et al, 2014). Thus, the observed increase in concentration of indoxyl sulfate and phenyl sulfate in this study support their previously described roles in modifying cytochrome P450 regulation in CKD (Fig. 4). Further, our in vitro studies using Huh7 human hepatoma cells showed indoxyl sulfate decreases CYP3A4 expression in a concentration dependent manner. Interestingly, of the metabolites found by correlation to Cyp3a2 or Cyp2c11, the 5 metabolites that are associated with cytochrome P450 downregulation in the literature are all gut-derived uremic toxins. Indoxyl sulfate is a potent aryl hydrocarbon receptor (AhR) agonist and has been shown to induce CYP1A1, CYP1A2, CYP1B1, UGT1A1, and UGT1A6 (Schroeder et al, 2010). Although AhR agonism is typically thought of with respect to enzyme induction, there is some evidence to suggest AhR agonism decreases Cyp3a and Cyp2c11 expression, which may be a mechanism for the effects we observed in our rat model of CKD (Shaban et al, 2005).

Indoxyl sulfate, phenyl sulfate, and 4-ethylphenyl sulfate concentration all increase after day 28 when changes are simultaneously observed in the gut microbiota that was phylogenetically analyzed using 16S sequencing. This lends support to the idea that uremia may be driving the change in gut microbial abundance through a damaged gut wall (Magnusson et al, 1991; Felizardo et al, 2016). The late and pronounced increase in gut-derived uremic toxins also suggests dysbiosis contributes to the exacerbation of uremia, likely adding to the uremic milieu by increasing the number of bacteria capable of uremic toxin production (Felizardo et al, 2016). Multivariate analysis showed the microbiota was most significantly influenced by time (ie, age) and secondarily by disease state. This correlation suggests that the microbiota changes caused by CKD induction are less profound than age-associated bacterial changes. Additionally, in comparison to the metabolomic PCA, microbial clustering with respect to disease state was poor. This may indicate that the uremic environment in the plasma and liver are altered prior to the onset of dysbiosis. In human subjects the *Firmicutes* to *Bacteroidetes* ratio is often associated with gut dysbiosis with an increased *Firmicutes* to *Bacteroidetes* ratio previously observed in CKD subjects compared with healthy controls (Liu et al, 2023). This is consistent with our observation of increased *Turicibacter* (from the phylum Firmicutes) and a decrease in *Parabacteroides* (from the phylum Bacteroidetes) in our rat model of CKD. Bacterial families significantly changed on days 28 and 42 contained strains capable of producing at least one of the following genes: urease, tryptophanase, phosphotransbutyrylase, or butyrate kinase, although the bacteria were inconsistently characteristic of either control or CKD rats (Wong et al, 2014). The sole bacterial genus significantly changed due to disease state prior to day 14 was of the order Clostridiales, in accordance with the findings of Barrios et al (2015) who sequenced the gut microbiota of 855 people and correlated bacteria from the order Clostridiales with early renal decline.

Although interesting to examine the temporal relationship for OTUs differing due to CKD, only 2 bacterial genera, *Turicibacter* and *Parabacteroides*, were significant on ≥2 consecutive days, best correlating with cytochrome P450 trends. *Turicibacter* was the most consistently changed bacteria, changing as early as day 14 through to day 42 with an increasing trend as CKD progressed. Identifying the genus *Turicibacter* in CKD animals is a novel finding. *Turicibacter* are gram-positive, strictly anaerobic, rod-shaped bacteria of which very little is known. *Turicibacter* has been identified in the blood of febrile patients with acute appendicitis (Bosshard et al, 2002) and associated with pouchitis—a complication of proctocolectomy—in ulcerative colitis patients (Falk et al, 2007). A fecal microbiota transplant from healthy human into colons of germ-free rats also identifies *Turicibacter* sp (Licht et al, 2007). Only 4 strains, within the *sanguinis* species*,* have been published to date: MOL361 (Bosshard et al, 2002), PC909 (Cuív et al, 2011), ZCY83 (Cao et al, 2015), and H121 (Auchtung et al, 2016). A BLASTn search of our *Turicibacter* sequence matched the MOL361 species with 100% identity (NR_028816.1). Assuming all rats were exposed to *Turicibacter* for the study duration, our findings suggest CKD animals are more susceptible to gut colonization by *Turicibacter*.

The BLASTn results for the *Parabacteroides* genus OTU suggested 99% sequence identity to 2 stains of the species *distasonis*: strain ATCC 8503 (NR_074376.1) and JCM 5825 (NR_041342.1). In 2006, *Bacteroides distasonis was reclassified as Parabacteroides distasonis* and thus, all subsequent information pertains to either classification (Sakamoto and Benno, 2006). *Parabacteroides* is a gram-negative, anaerobic, nonspore-forming genus. *P distasonis* is classified in the Kyoto Encyclopedia of Genes and Genomes pathway database as an opportunistic pathogen capable of anaerobic infection (Xu et al, 2007). Analysis of bacteria capable of generating phenol and indole compounds found *P distasonis* proficient at producing p-cresol (Gryp et al, 2017) and indoxyl sulfate (Zhang and Davies, 2016). In general, it seems *P distasonis* is potentially both harmful and beneficial depending on translocation and abundance. Our results show a unique trend where CKD rats have a stable level of *Parabacteroides* and controls slowly reduce the abundance of this genus after 28 days. Given the multitude of associations with disease, *Parabacteroides* may be taking advantage of the dysbiotic state in CKD when it is normally removed in controls by other healthy bacteria as a part of the progression in age-associated microbial changes.

It should be noted that our study has some limitations. We evaluated the impact of progressive CKD on the expression of Cyp3a2 and Cyp2c11 because they are known to metabolize a large fraction of marketed drugs and because previous studies have shown profound decreases in expression and activity in severe CKD. Cyp3a2 and Cyp2c11 are not the only P450s that are decreased in rat models of CKD. Other studies have shown Cyp1a2, Cyp2b6, and Cyp2d1/2 are also decreased in rat models of CKD (Michaud et al, 2005; Kucey et al, 2019). In addition, the uremic toxin indoxyl sulfate is known to regulate AhR mediated CYP1A1/1A2 expression (Schroeder et al, 2010). Future studies should evaluate the effect of progressive kidney disease on these and other P450s and delineate the role of indoxyl sulfate on a broader number of P450 enzymes. Another limitation that should be noted is that our study shows a temporal relationship between severe CKD and uremia, but does not demonstrate causality with respect to changes in P450 expression and activity. Our study was conducted on male rats so the findings may not be generalizable to females. Finally, it should be noted that there are differences in the regulation of rat and human P450 enzymes. Although there is some evidence that the pharmacokinetics of substrate drugs are altered in human patients with CKD, other studies show no difference between patients with CKD and subjects with normal kidney function (Dreisbach et al, 2003; Nolin et al, 2009; Thomson et al, 2015; Tuey et al, 2022). Similarly, we used Huh7 cell to investigate the impact of uremic toxins on CYP3A4 expression. While Huh7 cells are a good model to investigate changes in CYP3A4 expression, future studies should use primary human hepatocytes so the effect on other P450s (eg, CYP2C9) can be investigated. Confirmation that indoxyl sulfate causes a corresponding decrease in CYP3A4 activity and protein level along with investigation of nuclear receptor activity would help to clarify the mechanism of this finding.

In conclusion, our study clearly demonstrates that Cyp3a2 and Cyp2c11 expression and activity are decreased very shortly (as early as 3 days) after initiation of CKD induced by adenine. The early detection of cytochrome P450 downregulation and later surge of gut-derived uremic toxin concentrations suggest other factors are involved in cytochrome P450 regulation in early stages of CKD. A temporal association was established between severe CKD, cecal dysbiosis and increase in gut-derived uremic toxins indoxyl sulfate, phenyl sulfate, and 4-ethylphenyl sulfate. This association may suggest a positive-feedback loop of uremia and dysbiosis suspected to drive severe CKD.

## Conflict of interest

The authors declare no conflicts of interest.
